# Trends in Blood Pressure Control in US Adult CKD Patients from 1999 to 2018

**DOI:** 10.7150/ijms.103107

**Published:** 2025-01-13

**Authors:** Anbang Sun, Lin Zhu, Yanmin Xia, Yao Zheng, Bicheng Hu, Fanghua Li, Yanhong Liao

**Affiliations:** 1 Department of Transfusion Medicine, Traditional Chinese and Western Medicine Hospital of Wuhan, Tongji Medical College, Huazhong University of Science and Technology, Wuhan, 430030, Hubei, China.; 2 Department of Anatomy, Tongji Medical College, Huazhong University of Science and Technology, Wuhan, 430030, Hubei, China.; 3 Department of Nephrology, The First Affiliated Hospital of Zhengzhou University, Zhengzhou, 450052, Henan, China.; 4 Key Laboratory of Neurological Diseases of Ministry of Education, Tongji Medical College, Huazhong University of Science and Technology, Wuhan 430030, China.

**Keywords:** chronic kidney disease, high blood pressure, hypertension, clinical practice guidelines

## Abstract

**Background:** Blood pressure (BP) control can slow down the progression of chronic kidney disease (CKD) and protect against cardiovascular diseases, significantly improving patient survival. Herein, we analyzed the changes in BP control in adult CKD patients with hypertension in the United States from 1999-2000 to 2017-2018.

**Methods:** National Health and Nutrition Examination Survey (NHANES) data from 1999-2000 to 2017-2018 were analyzed, including 5,510 adult CKD patients with BP above 140/90 mmHg or those under an antihypertensive regimen.

**Results:** The proportion of adult CKD patients with uncontrolled BP decreased from 72.9% in 1999-2000 to 46.6% in 2013-2014, then increased to 56.9% in 2017-2018. Although adult CKD patients with albumin-creatinine rate (ACR) 30-299 mg/g or ACR ≥300 mg/g were more likely to take antihypertensive medication than those with ACR <30 mg/g (PR: 2.76, 95% CI: 1.63-4.79 and PR: 4.59, 95% CI: 2.37-9.51), they were more likely to have uncontrolled BP than those with ACR <30 mg/g ((multivariable-adjusted prevalence ratio (PR): 2.25, 95% CI: 1.39-3.75 and PR: 3.14, 95% CI: 1.71-6.07). Adult CKD patients (eGFR ≥60 mL/min/1.73m^2^) being aware of their high BP diagnosis were less likely to take antihypertensive medication than those with eGFR 30-59 mL/min/1.73m^2^ (PR: 0.27, 95% CI: 0.09-0.65).

**Conclusions:** These results show that BP control should be reinforced in adult CKD patients, particularly in those with ACR ≥300 mg/g, while patients with eGFR ≥60 mL/min/1.73m^2^ should enhance awareness of taking antihypertensive medication.

## Introduction

Chronic kidney disease (CKD) defined by glomerular filtration rate (GFR) below 60 mL/min/1.73m^2^, or proteinuria, or markers of renal injury (hematuria or morphology abnormalities) lasting for at least 3 months 1-4. CKD and hypertension are common diseases that interact and associate with each other in the general population 5, 6. On the one hand, when the estimated glomerular filtration rate (eGFR) decreases, the morbidity and severity of hypertension increase 7. On the other hand, controlled blood pressure (BP) can slow the progression of CKD from stage 3 or stage 4 to end stage of renal disease (ESRD) and reduce the incidence of cardiovascular disease (CVD) 8-10. In addition, CKD and hypertension are independent risk factors for CVD 11-14. Moreover, CVD incidence and mortality risk increase when CKD and hypertension are concomitant 15. Between 1999-2000 and 2007-2008, the percentage of US adults with controlled blood pressure (<140/90 mmHg) increased, then stabilized between 2009-2010 and 2013-2014, and then declined 16.

In this analysis, we aim to examine whether the BP control rates in CKD patients with hypertension improved in the United States from 1999-2000 to 2017-2018. Additionally, the trends of antihypertensive medication use and hypertension awareness were analyzed. Moreover, we identified the subgroups of individuals with CKD and albuminuria whose BP control needs to be strengthened.

## Methods

### Data source

National Center for Health Statistics (NCHS) conducts National Health and Nutrition Examination Survey (NHANES) using a stratified, multistage probabilistic sampling approach. Consequently, the study is representative of the noninstitutionalized population in the US. The survey in NHANES is cross-sectional and has been conducted in every two-year cycle, and we pooled 10 cycles from 1999-2000 to 2017-2018. All the data used in this study were downloaded from NHANES. NHANES was approved by the Research Ethics Review Board of the National Center for Health Statistics (NCHS) and all participants of NHANES gave written informed consent prior to any data being collected (https://www.cdc.gov/nchs/nhanes/irba98.htm).

### Data collection

NHANES data includes interviews conducted in-home, physical examinations and laboratory measurements on blood and urine samples in a mobile examination center. The covariates included in this study, such as age, race/ethnicity, sex, education, health insurance, health care, body mass index (BMI), smoking status, and diabetes along with their assessment methods, are listed in Supplementary Table 1.

### Study population

For our analytic population, we included participants with completed serum creatine, urinary albumin and creatinine measurements (n=63,970). According to the Chronic Kidney Disease Epidemiology Collaboration equation (eGFR = 141 × min (Scr/κ, 1)^α^ × max (Scr/κ, 1)^-1.209^ × 0.993^Age^ × 1.018 [if female] × 1.159 [if Black], Scr stands for serum creatinine, with κ values of 0.7 for women and 0.9 for men, α values of -0.329 for women and -0.411 for men, min representing the minimum of Scr/κ or 1, and max representing the maximum of Scr/κ or 1.) 17, 18, CKD was defined as eGFR <60 mL/min/1.73m^2^, or as urine albumin-creatinine rate (ACR) ≥30 mg/g 17. A total of 10,684 participants met the CKD diagnostic criteria. We excluded those who were less than 20 years old or were pregnant, resulting in 8,876 participants. Among them, 7,710 patients had three independent systolic blood pressure (SBP) and diastolic blood pressure (DBP) measurements. We included those who had SBP ≥140 mmHg or DBP ≥90 mmHg, or answered positively to “Are you now taking prescribed medicine for high BP?” Finally, a total of 5,510 participants were enrolled in this current analysis. Supplementary Figure 1 details the selection of the study sample for this analysis.

### Blood pressure (BP) measurement

Trained physicians performed BP measurements-according to a standardized protocol 19. After a 5-minute rest, three measurements of BP were performed by a mercury sphygmomanometer and an appropriate cuff size at 30-second intervals. If the BP measurement was incomplete or interrupted, a fourth BP measurement was taken. The mean SBP and DBP were calculated using three measurements in each participant.

### Hypertension awareness and antihypertensive medication use

A positive response to the question “Have you ever been told by a doctor or other health care professional that you have hypertension?” was considered hypertension awareness 16. The information on antihypertensive medication use was obtained from the questionnaire “Are you now taking prescribed medication for high BP?” 20.

### Recommended BP control targets

SBP <120 mmHg was the BP goal recommended by the 2024 KDIGO guideline 1. For CKD patients with ACR ≥30 mg/g, SBP ≤130 mmHg and DBP ≤80 mmHg was recommended by the 2012 KDIGO guideline, while SBP ≤140 mmHg and DBP ≤90 mmHg was recommended for those patients with no albuminuria (ACR <30 mg/g) 21. The 2017 American College of Cardiology (ACC)/ American Heart Association (AHA) guideline recommended SBP/DBP **<**130/80 mmHg for all CKD patients 22. In contrast, the 2014 Eighth Joint National Committee (JNC 8) guideline recommended SBP/DBP **<**140/90 mmHg for all CKD patients 23.

### Analysis

The characteristics of CKD patients with hypertension in the US, such as demographics, socioeconomic, health care, and risk factors (diabetes, obesity, smoking) 24-26, were analyzed across the ten two-year cycles from 1999-2000 to 2017-2018. We also assessed the mean SBP and DBP and the distribution of BP categories among adult CKD patients with high BP. The proportion of uncontrolled BP were analyzed for all adult CKD patients with high BP and those using antihypertensive medication. The proportions were assessed within different subgroups, including age, sex, race/ethnicity, education, household income, health insurance, health care, eGFR, ACR, diabetes, obesity, and smoking status. Join-point Regression Program 4.9.1.0 software (Statistical Research and Applications Branch, National Cancer Institute) was used to analyze changes in trends for estimated proportion of the uncontrolled BP among adult CKD patients and separately among those being aware of their hypertension or among those taking antihypertensive medication.

Affected by COVID-19, 2015-2018 is the latest volunteer data of the two calendars, so the population of these two calendars is selected for retrospective analysis. Data from 2015-2018 were pooled to examine factors associated with uncontrolled BP among all adult CKD patients with high BP and adult CKD patients taking antihypertensive medication using logistic and multinomial logistic regression models. Factors associated with hypertension awareness among all adult CKD patients, and antihypertensive medication use among adult CKD patients being aware of they had high BP were estimated using logistic and multinomial logistic regression models. Missing data were multiply imputed using logistic regression. For categorical variables, we used logistic regression for multiple imputation. Specifically, logistic regression was used for binary results variables, and ordinal logistic regression was used for ordinal categorical variables with more than two levels. Linear regression is used for multiple imputation of continuous variables. There is a small amount of missing data from NHANES including education level (0.2%), income (6.5%), ACR (2.5%), health insurance (0.3%), times receive healthcare over past year (0.2%), and BMI (3.1%). The number of imputations conducted in this analysis was 20 27, 28.

All analyses were conducted based on NHANES survey weights, strata, and complex sampling design units. R (R Project for Statistical Computing, https://www.r-project.org/, version 4.1.3) was used in all analyses, and p <0.05 was considered statistically significant.

## Results

### Characteristics of the study participants

This analysis showed that the distribution of age, sex, health care, and smoking status of adult CKD participants with hypertension in NHANES were unchanged from 1999 to 2018, while the percentage of other race/ethnicity (including other Hispanic, other multi-racial), high school and college degrees, higher income (more than $45,000), health insurance (including private, Medicare, and government health insurance), diabetes, obesity, and CKD (eGFR 30-59 mL/min/1.73m^2^) with no albuminuria increased. Table 1 details the characteristics of the participants.

### Distribution of BP among adult CKD with hypertension

**Figure 1:**
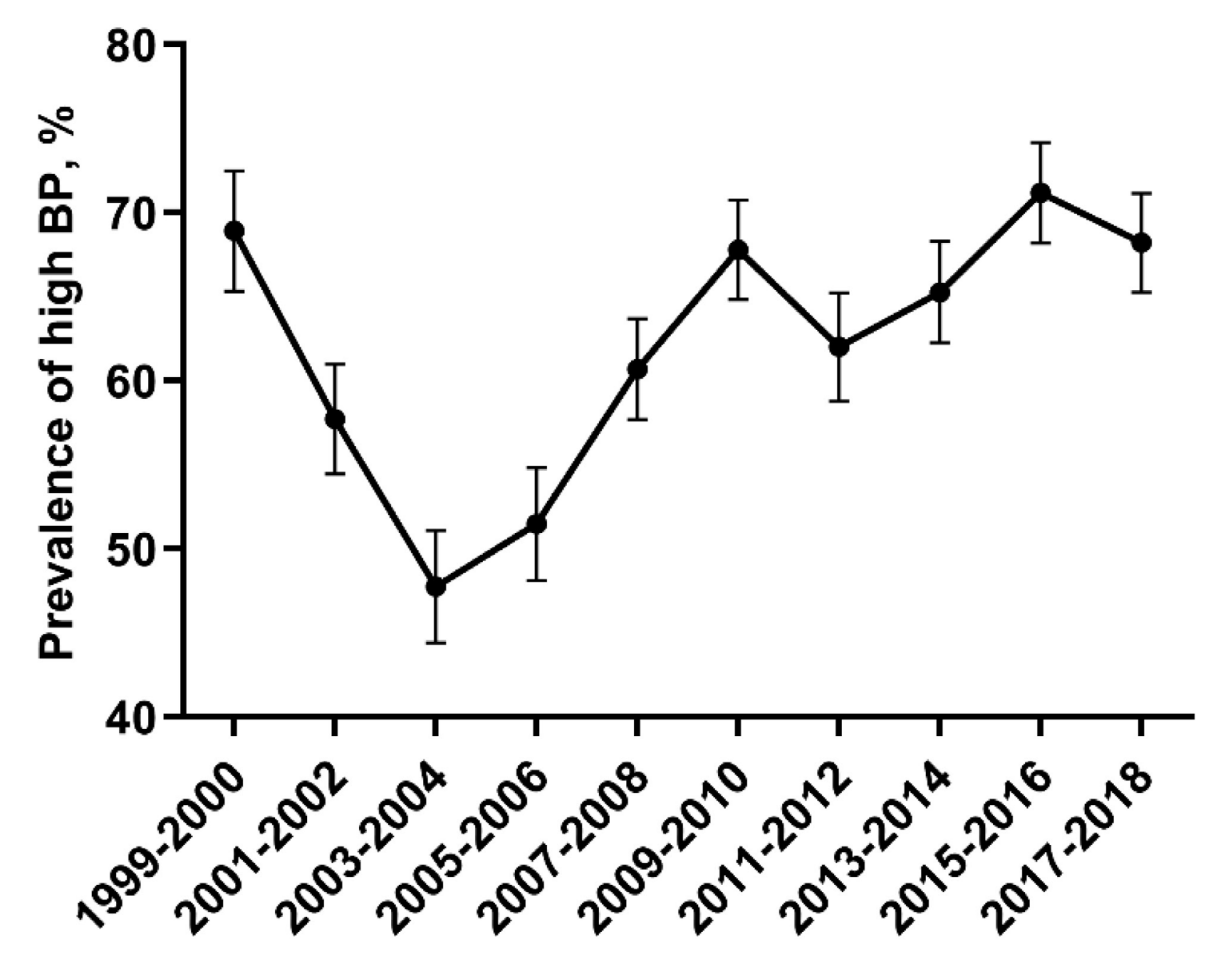
** Percentage of adult CKD with high BP.** The percentage of adult CKD participants with high BP decreased from 1999-2000 through 2003-2004, and increased from 2005-2006 through 2017-2018. Error bars indicate 95% confidence intervals. Hypertension was defined by systolic blood pressure ≥140 mmHg and/or diastolic blood pressure ≥90 mmHg and/or antihypertensive medication use. Abbreviations: BP, blood pressure.

The percentage of adult CKD participants with high BP decreased from 1999-2000 through 2003-2004, and increased from 2005-2006 through 2017-2018 (Figure 1). As presented in Table 2, the most common category of SBP/DBP from 1999-2000 through 2017-2018 was 140-159/90-99, with the proportion decreasing from 1999 to 2014, and increasing to the same level as 2005-2006.

**Figure 2:**
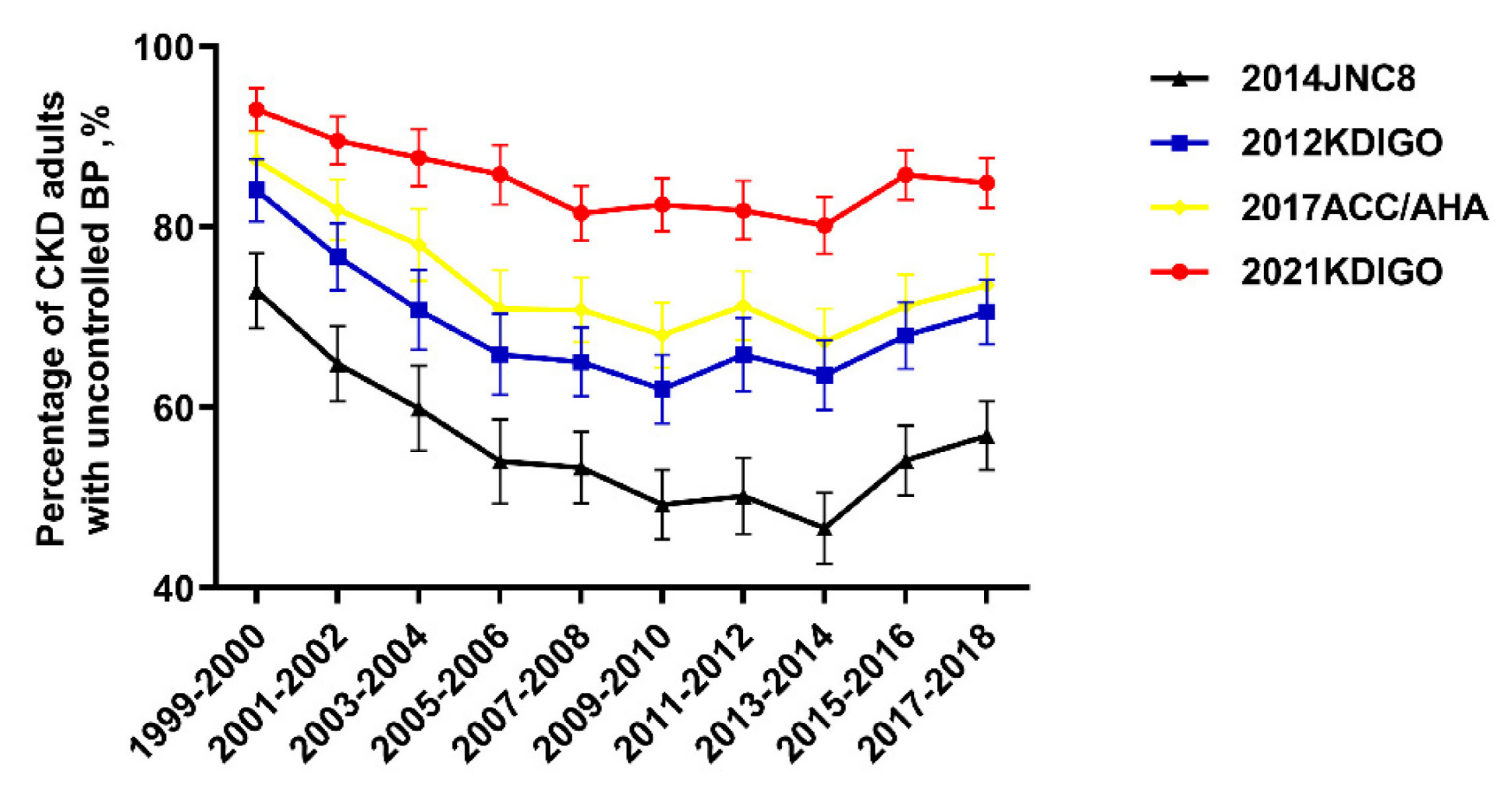
**Percentage of CKD adults with uncontrolled BP according to different guidelines.** Trends for uncontrolled BP among adult CKD participants with high BP were nonlinear, with an inflection point around 2013-2014. Error bars indicate 95% confidence intervals. Abbreviations: ACC: American College of Cardiology; AHA: American Heart Association; JNC8: the Eighth Joint National Committee; KDIGO: Kidney Disease Improving Global Outcomes.

Trends for uncontrolled BP among adult CKD participants with high BP were nonlinear, with an inflection point around 2013-2014 (Figure 2). The percentage of uncontrolled BP among CKD patients with high BP decreased from 72.9% (95% confidence interval (CI), 68.8 to 77.1) in 1999-2000 to 46.6% (95% CI, 42.7 to 50.5) in 2013-2014, then increased to 56.9% (95% CI, 53.1 to 60.7) in 2017-2018 (Table 3). When adjusted for demographic, socioeconomic, and clinical characteristics, adult CKD patients with ACR 30-299 mg/g were more likely to have uncontrolled BP compared to patients with no albuminuria (multivariable-adjusted prevalence ratio (PR): 2.25, 95% CI: 1.39-3.75, left panel of Table 4). Similarly, CKD patients aged more than 75 years compared to 20-44 years (PR: 2.55, 95% CI: 1.07-5.84) were more likely to suffer from uncontrolled BP. Uncontrolled BP was less likely among those who had healthcare visit in past year compared with those who had no healthcare visit in past year (PR: 0.29, 95% CI: 0.15-0.51). Adult CKD patients with obese were less likely to have uncontrolled BP when compared with normal weighted adult CKD patients (PR: 0.70, CI: 0.49-0.99).

**Figure 3:**
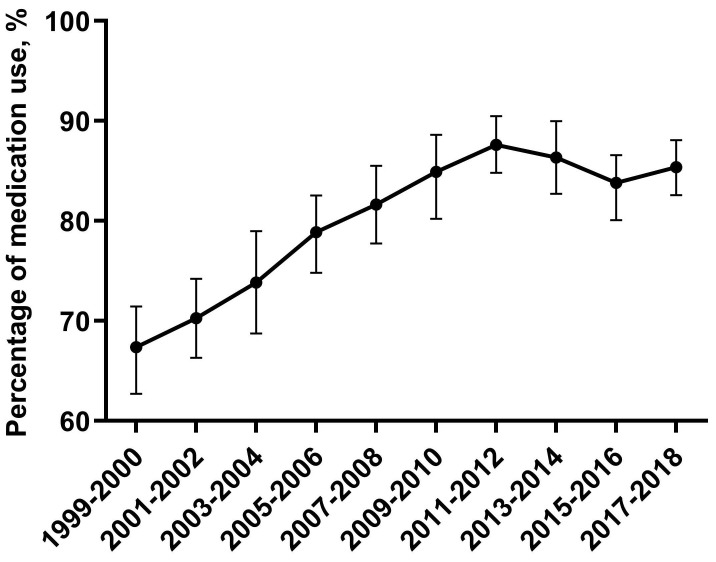
**Trends of CKD adults with hypertension taking antihypertensive medication.** Trends for adult CKD participants taking antihypertensive medication were nonlinear, with an inflection point around 2011-2012. Error bars indicate 95% confidence intervals.

Trends for adult CKD participants taking antihypertensive medication were nonlinear, with an inflection point around 2011-2012 (Figure 3). The percentage of adult CKD patients taking antihypertensive medication among adult CKD patients with high BP increased from 67.0% (95% CI, 62.7-71.4) in 1999-2000 to 87.6% (95% CI, 84.8-90.4) in 2011-2012, then decreased to 85.3% (95% CI, 82.6-88.0) in 2017-2018 (Figure 3). Among adult CKD patients taking antihypertensive medication, those who aged above 75 vs 20-44 (PR: 3.43, 95% CI: 1.81-6.61) were more likely to have uncontrolled BP. When compared with Non-Hispanic White, Non-Hispanic Black taking antihypertensive medication were more likely to have uncontrolled BP (PR: 1.41, 95% CI: 1.00-1.98). Uncontrolled BP were more common among adult CKD patients taking antihypertensive medication with ACR 30-299 mg/g vs ACR <30mg/g (PR: 3.05, 95% CI: 2.03-4.62).

Moreover, patients with house income more than 75,000$ taking antihypertensive medication were less likely to have uncontrolled BP in comparison to those with house income less than 44,999$ (PR: 0.63, 95% CI: 0.43-0.91). In addition, those had healthcare visit in past year taking antihypertensive medication were less likely to have uncontrolled BP (PR: 0.36, 95% CI: 0.08-1.10) (right panel of Table 4).

Trends for adult CKD patients being aware of their hypertension diagnosis were nonlinear, with an inflection point around 2011-2012 (Supplementary Figure 2A). The percentage of adult CKD patients being aware of their hypertension diagnosis among adult CKD patients with high BP increased from 75.2% (95% CI, 71.2-79.2) in 1999-2000 to 90.2% (95% CI, 87.7-92.7) in 2011-2012, then decreased to 87.9% (95% CI, 85.4-90.4) in 2017-2018 (Supplementary Figure 2A). After adjustment for multivariable, hypertension awareness was more common among those who had healthcare visit in the past year vs. those who had no healthcare visits in the past year (PR: 3.73, 95% CI: 2.07-6.71). Hypertension awareness was more prevalent among obese patients vs. normal weighted patients (PR: 1.70, 95% CI: 1.03-2.78), and among those current smoked vs. never smoked (PR: 2.70, 95% CI: 1.48-5.21), as well as among those who with eGFR ≤29 mL/min/1.73m^2^ vs. eGFR 30-59 mL/min/1.73m^2^ (PR: 5.26, 95% CI: 1.29-11.57).

Trends for adult CKD patients who were aware of their hypertension taking antihypertensive medication were nonlinear, with an inflection point around 2011-2012, stabilizing thereafter (Supplementary Figure 2B). The percentage of adult CKD patients being aware of their hypertension taking antihypertensive medication increased from 89.2% (95% CI, 85.9-92.5) in 1999-2000 to 97.1% (95% CI, 95.6-98.1) in 2011-2012, and 97.0% (95% CI, 95.7-98.4) in 2017-2018 (Supplementary Figure 2B). Among adult CKD patients being aware of their high BP diagnosis, those over 75 years old were more likely to take antihypertensive medication vs. patients aged 20-44 (PR: 7.90, 95% CI: 2.05-15.3, right panel of Supplementary Table 2). Among adult CKD patients being aware of their high BP diagnosis, antihypertensive medication use was less likely among those who had government health insurance vs. those who had no health insurance (PR: 0.34, CI: 0.14-0.86). In addition, adult CKD patients (eGFR ≥60 mL/min/1.73m^2^) being aware of their high BP diagnosis were less likely to take antihypertensive medication vs. those who had eGFR 30-59 mL/min/1.73m^2^ (PR: 0.27, 95% CI: 0.09-0.65).

## Discussion

In this analysis, the estimated percentage of uncontrolled BP among adults with CKD declined from 1999-2000 through 2013-2014, and then increased from 2015-2016. This is in line with the NCHS results and a previous study which found that the percentage of controlled BP decreased from 2013-2014 among adults with hypertension in the US 16, 29.

In 2003, the Seventh Joint National Committee (JNC 7) guideline recommended BP control targets of SBP <130 mmHg and DBP < 80 mmHg for adult CKD 20. The Action to Control Cardiovascular Risk in Diabetes trial (ACCORD) in 2010 demonstrated that intensive BP control (SBP<120 mm Hg vs <140 mm Hg) in adult diabetes patients did not lower the risk of CVD mortality 30. JNC 8 guideline recommended a higher BP control target of SBP < 140 mmHg and DBP < 90 mmHg for adult CKD in 2014 23. The uncontrolled BP increased from 2013-2014, whether according to the BP control target of the 2021 KDIGO guideline, or the 2012 KDIGO guideline, or the JNC 8 guideline 21-23, 31. These results suggest that the treatment of hypertension has generally shifted to less intensity.

Although uncontrolled BP among adult CKD patients has decreased since 1999-2000, the subgroups with elderly adults (age ≥75 years old), and ACR 30-299 mg/g had higher uncontrolled BP rates. Even taking antihypertensive medication, those aged more than 75 years old, ACR 30-299 mg/g, or Non-Hispanic Black were more likely to have uncontrolled BP. These results were in line with Chronic Renal Insufficiency Cohort (CRIC) Study 7. Other study indicated that refractory hypertension was associated with elderly patient, black race, and also albuminuria 32.

This current study showed that adult CKD patients being aware of their hypertension increased from 75.2% in 1999-2000 to 87.9% in 2017-2018. Hypertension awareness was more prevalent among obese, current smoked or those who with eGFR ≤29 mL/min/1.73m^2^. Not all patients being aware of their high BP take antihypertensive medication, those with eGFR ≥60 mL/min/1.73m^2^ were less likely to take antihypertensive medication when compared with those who had eGFR 30-59 mL/min/1.73m^2^ in this analysis. Prior to 2010, a survey from the National Kidney Foundation's Kidney Early Evaluation Program (KEEP) found that 86.2% of adult CKD patients had hypertension, with an awareness rate of 80.2% and a medication usage rate of 70.0%, but only 13.2% had their BP under control. They also found that those with eGFR of 30-59 mL/min/1.73m^2^ were more likely to have controlled BP than patients with eGFR ≥60 mL/min/1.73m^2^ 33. Data from China showed that hypertension awareness, treatment and control rate in adults with CKD in 2004-2005 were 87.2%, 85.9%, and 30.0%, respectively 34. Awareness of BP control should be reinforced to ensure that adult CKD patients, especially those with eGFR ≥60 mL/min/1.73m^2^ are screened for hypertension and that antihypertensive treatment is administered promptly.

As an important marker of renal damage, albuminuria has an independent correlation with CKD progression and CVD incidence 15, 35. Hypertensive patients with ACR ≥30 mg/g were more likely to develop moderate-to-severe intracranial atherosclerotic stenosis 36. There was a higher prevalence of microvascular disease in elderly hypertensive male patients without comorbidities with a higher ACR 37. BP control reduces albuminuria, which slows the progression of CKD and reduces the incidence of CVD 38. Intensive BP control (SBP <120 mmHg) has a more reno-protective effect in those with albuminuria (>1 g/day) than in those without albuminuria, signifying that intensive BP control could possibly reduce albuminuria 30, 39, 40. In the current analysis, although adult CKD with ACR 30-299 mg/g were more likely to take antihypertensive medication when they were aware of their high BP, this subgroup was more likely to have uncontrolled BP. While those with eGFR ≥60 mL/min/1.73m^2^ were less likely to take antihypertensive medication. As a result, effort should be made to increase antihypertensive medication use in adult CKD with eGFR ≥60 mL/min/1.73m^2^, and strengthen BP control in those with ACR 30-299mg/g.

In the current analysis, elderly CKD patients (age ≥75 years) were more likely to have BP above the recommended target, even among those taking antihypertensive medication. Hypertension was prevalent in 82.0% and treated in 87.3% of elderly CKD patients in China, but only 29.6% of those with BP <140/90 mmHg and 12.1% of BP <130/80 mmHg 41. Another American study revealed that CKD patients aged ≥80 years had higher mean SBP when compared with those aged 40-64 or 65-79 years old 42. An intensive BP control (SBP=120 mmHg) among those over 75 years old was effective in the SPRINT (the Systolic Blood Pressure Intervention Trial) subgroup analysis, with reduced cardiovascular events and no increase in adverse events 43. Taken together, our findings show that doctors and patients should strengthen BP control in elderly CKD patients.

The strength of the current study is that the patient population from the NHANES database is representative of the US population as a whole. Moreover, NHANES enrolled a large sample size and followed standardized laboratory measurement and examination procedures. Furthermore, our present study analyzed over 20 years of data about CKD patients with high BP to analyze trends in BP control. Nevertheless, our analysis also has several limitations. Firstly, clinical guidelines define CKD as a decrease in eGFR or the presence of proteinuria for at least three months 20. However, we may have misclassified CKD as we relied on only a single measurement for serum creatinine and urinary albumin/creatinine from NHANES. However, Equations based on creatinine often overestimated GFR in Black individuals and produced varied outcomes in non-Black individuals. They are also had greater bias when GFR exceeds 60 mL/min/1.73 m^2^ and lack of precision in population predictions for major CKD-related conditions like diabetes. Cystatin C-based equations exhibited minimal biases and might be better suited for predicting kidney function of Black individuals 26, 44. Besides, Serum creatinine has additional shortcomings that can affect its reliability in eGFR. For instance, in older patients with reduced muscle mass, serum creatinine levels may be low, leading to an overestimation of GFR 45-47. Hormones such as thyroid hormones and glucocorticoids can influence muscle metabolism and creatinine production, thereby affecting serum creatinine concentrations 48, 49. Dehydration can lead to a reduction in plasma volume, which may result in an apparent increase in serum creatinine concentration, falsely suggesting a decline in renal function 50. There are also other factors can affect serum creatinine levels, further complicating the estimation of GFR, such as variability in measurement methods 51, inulin clearance or not 52, dietary influence (e.g., high-protein diet) 53-55. Thus, the more efficiency and accurate methods or equations are needed to be explored for kidney function determination. Secondly, antihypertensive medication use was ascertained from participant reports, which could be incorrectly documented or may be biased from clinical practice. Due to the positive response to the question “Have you ever been told by a doctor or other health care professional that you have hypertension?”, the prevalence of hypertension may be overestimated. As convinced by other researchers, compared with pharmacy records, self-reported antihypertensive medication showed better sensitivity, positive predictive value and specificity 56. Self-reported medication use is accurate compared with prescription data, especially for medication classes that are clearly defined 57. Hence, self-reported antihypertensive medication errors by patients do not significantly affect hypertension prevalence.

The proportion of uncontrolled BP in US adult CKD patients decreased from 1999-2000 to 2013-2014, but increased after that. Collectively, our findings reveal that BP control should be reinforced in adult CKD patients, particularly in those with ACR 30/-299 mg/g.

## Supplementary Material

Supplementary figures and tables.

## Figures and Tables

**Table 1:** Characteristics of adult CKD with high BP from 1999-2000 to 2017-2018.

Characteristic	Calendar period									
	1999-2000 (n=443) (95% CI)	2001-2002 (n=509) (95% CI)	2003-2004 (n=414) (95% CI)	2005-2006 (n=437) (95% CI)	2007-2008 (n=617) (95% CI)	2009-2010 (n=644) (95% CI)	2011-2012 (n=540) (95% CI)	2013-2014 (n=620) (95% CI)	2015-2016 (n=632) (95% CI)	2017-2018 (n=654) (95% CI)
Age, y (%)										
20-44	8.4(5.8-10.9)	7.5(5.2-9.7)	5.6(3.3-7.8)	8.2(5.7-10.8)	5.8(4.0-7.7)	6.2(4.3-8.1)	7.8(5.5-10.0)	7.3(5.2-9.3)	8.5(6.4-10.7)	5.7(3.9-7.4)
45-64	22.8(18.9-26.7)	23.4(19.7-27.1)	24.2(20.0-28.3)	26.1(22.0-30.2)	31.0(27.3-34.6)	25.3(22.0-28.7)	27.2(23.5-31.0)	32.3(28.6-35.9)	30.4(26.8-34.0)	31.2(27.6-34.7)
65-74	28.4(24.2-32.6)	23.8(20.1-27.5)	28.3(23.9-32.6)	25.4(21.3-29.5)	26.6(23.1-30.1)	27.6(24.2-31.1)	25.9(22.2-29.6)	27.1(23.6-30.6)	25.2(21.8-28.5)	27.2(23.8-30.6)
≥75	40.4(35.8-45.0)	45.4(41.1-49.7)	42.0(37.3-46.8)	40.3(35.7-44.9)	36.6(32.8-40.4)	40.8(37.0-44.6)	39.1(35.0-43.2)	33.4(29.7-37.1)	35.9(32.2-39.7)	35.9(32.3-39.6)
Gender (%)										
Female	50.8(46.1-55.4)	49.545.2-53.9)	55.3(50.5-60.1)	49.0(44.3-53.7)	54.3(50.4-58.2)	50.5(46.6-54.3)	49.4(45.2-53.7)	51.9(48.0-55.9)	51.7(47.8-55.6)	48.6(44.8-52.5)
Race/ethnicity (%)										
Non-Hispanic White	41.1(36.5-45.7)	59.5(55.3-63.8)	56.8(52.0-61.5)	55.6(50.9-60.3)	51.2(47.3-55.2)	51.1(47.2-54.9)	41.9(37.7-46.0)	49.8(45.9-53.8)	38.1(34.3-41.9)	43.3(39.5-47.1)
Non-Hispanic Black	23.7(19.7-27.7)	22.4(18.8-26.0)	17.9(14.2-21.6)	28.6(24.4-32.8)	24.5(21.1-27.9)	21.0(17.8-24.1)	33.7(29.7-37.7)	25.0(21.6-28.4)	23.9(20.6-27.2)	25.8(22.5-29.2)
Mexican	27.5(23.4-31.7)	13.4(10.4-16.3)	18.8(15.1-22.6)	11.2(8.3-14.2)	13.1(10.5-15.8)	15.7(12.9-18.5)	5.6(3.6-7.5)	10.3(7.9-12.7)	17.2(14.3-20.2)	8.4(6.3-10.5)
Other	7.7(5.2-10.2)	4.7(2.9-6.6)	6.5(4.1-8.9)	4.6(2.6-6.5)	11.2(8.7-13.7)	12.3(9.7-14.8)	18.9(15.6-22.2)	14.8(12.0-17.6)	20.7(17.6-23.9)	22.5(19.3-25.7)
Education (%)										
<High school	54.2(49.5-58.8)	40.7(36.4-44.9)	38.4(33.7-43.1)	39.1(34.6-43.7)	40.0(36.2-43.9)	35.7(32.0-39.4)	36.9(32.8-40.9)	29.2(25.6-32.8)	30.7(27.1-34.3)	23.5(20.3-26.8)
High school graduate and some college	37.7(33.2-42.2)	45.2(40.9-49.5)	49.8(44.9-54.6)	48.5(43.8-53.2)	46.5(42.6-50.5)	50.2(46.3-54.0)	49.4(45.2-53.7)	52.4(48.5-56.4)	54.4(50.5-58.3)	58.6(54.8-62.3)
College graduate	8.1(5.6-10.7)	14.1(11.1-17.2)	11.8(8.7-14.9)	12.4(9.3-15.4)	13.3(10.6-16.0)	14.1(11.4-16.8)	13.7(10.8-16.6)	18.4(15.3-21.4)	14.9(12.1-17.6)	17.9(15.0-20.8)
Household income, $ (%)										
<44999	79.7(75.9-83.4)	70.9(67.0-74.9)	73.9(69.7-78.1)	69.8(65.5-74.1)	72.3(68.8-75.8)	66.8(63.1-70.4)	71.1(67.3-74.9)	63.4(59.6-67.2)	69.5(65.9-73.1)	65.0(61.3-68.6)
45000-74999	10.8(7.9-13.7)	17.3(14.0-20.6)	16.2(12.6-19.7)	16.7(13.2-20.2)	15.2(12.4-18.1)	19.1(16.1-22.1)	13.7(10.8-16.6)	18.4(15.3-21.4)	16.6(13.7-19.5)	15.0(12.2-17.7)
≥75000	9.5(6.8-12.2)	11.8(9.0-14.6)	9.9(7.0-12.8)	13.5(10.3-16.7)	8.3(6.1-10.4)	12.3(9.7-14.8)	15.2(12.2-18.2)	18.2(15.2-21.3)	13.9(11.2-16.6)	20.0(17.0-23.1)
Type of health insurance (%)										
None	10.4(7.5-13.2)	6.7(4.5-8.8)	6.0(3.7-8.3)	10.8(7.9-13.7)	10.4(8.0-12.8)	11.0(8.6-13.4)	11.7(9.0-14.4)	9.8(7.5-12.2)	10.9(8.5-13.3)	8.1(6.0-10.2)
Private	58.9(54.3-63.5)	57.2(52.9-61.5)	53.1(48.3-57.9)	91.5(88.9-94.1)	92.4(90.3-94.5)	94.7(93.0-96.4)	90.6(88.1-93.0)	91.8(89.6-93.9)	89.7(87.3-92.1)	92.4(90.3-94.4)
Medicare	69.5(65.2-73.8)	72.3(68.4-76.2)	75.8(71.7-80.0)	96.8(95.1-98.4)	96.1(94.6-97.6)	97.0(95.7-98.4)	94.6(92.7-96.5)	96.0(94.4-97.5)	95.6(94.0-97.2)	95.3(93.6-96.9)
government	7.0(4.6-9.4)	9.8(7.2-12.4)	10.1(7.2-13.1)	40.7(36.1-45.3)	38.1(34.3-41.9)	37.4(33.7-41.2)	38.0(33.9-42.1)	40.5(36.6-44.3)	43.5(39.6-47.4)	43.0(39.2-46.8)
Had usual health care facility (%)	93.0(90.6-95.4)	97.1(95.6-98.5)	95.4(93.4-97.4)	94.5(92.4-96.6)	95.1(93.4-96.8)	94.6(92.8-96.3)	93.9(91.9-95.9)	95.5(93.8-97.1)	90.8(88.6-93.1)	92.7(90.7-94.7)
Had health care visit in past year (%)	92.8(90.4-95.2)	94.1(92.1-96.2)	94.9(92.8-97.0)	94.7(92.6-96.8)	94.2(92.3-96.0)	94.4(92.6-96.2)	92.4(90.2-94.6)	95.2(93.5-96.9)	91.5(89.3-93.6)	93.6(91.7-95.5)
Diabetes (%)	37.0(32.5-41.5)	35.4(31.2-39.5)	35.5(30.9-40.1)	38.0(33.4-42.5)	42.0(38.1-45.9)	42.9(39.0-46.7)	45.0(40.8-49.2)	41.8(37.9-45.7)	43.7(39.8-47.5)	45.7 (41.9-49.5)
Weight (%)										
Normal	22.6(18.7-26.5)	24.2(20.4-27.9)	24.6(20.5-28.8)	19.7(16.0-23.4)	22.9(19.5-26.2)	20.3(17.2-23.5)	21.1(17.7-24.6)	21.5(18.2-24.7)	17.9(14.9-20.9)	17.3(14.4-20.2)
Overweight	34.3(29.9-38.7)	38.1(33.9-42.3)	36.2(31.6-40.9)	34.6(30.1-39.0)	31.4(27.8-35.1)	32.5(28.8-36.1)	27.6(23.8-31.4)	31.0(27.3-34.6)	34.3(30.6-38.0)	30.9(27.3-34.4)
Obese	43.1(38.5-47.7)	37.7(33.5-41.9)	39.1(34.4-43.8)	45.8(41.1-50.4)	45.7(41.8-49.6)	47.2(43.3-51.1)	51.3(47.1-55.5)	47.6(43.6-51.5)	47.8(43.9-51.7)	51.8(48.0-55.7)
Smoking (%)										
Never smoked	48.5(43.9-53.2)	54.6(50.3-58.9)	54.3(49.5-59.1)	54.0(49.3-58.7)	52.5(48.6-56.5)	51.6(47.7-55.4)	54.1(49.9-58.3)	50.5(46.5-54.4)	50.5(46.6-54.4)	51.1(47.2-54.9)
Former smoker	35.4(31.0-39.9)	39.9(35.6-44.1)	39.9(35.1-44.6)	37.3(32.8-41.8)	35.7(31.9-39.4)	38.5(34.8-42.3)	36.3(32.2-40.4)	32.4(28.7-36.1)	33.4(29.7-37.1)	36.5(32.9-40.2)
Current smoker	13.1(10.0-16.2)	14.7(11.7-17.8)	14.5(11.1-17.9)	16.7(13.2-20.2)	16.9(13.9-19.8)	13.0(10.4-15.6)	17.8(14.6-21.0)	18.1(15.0-21.1)	17.1(14.2-20.0)	14.5(11.8-17.2)
eGFR (mL/min/1.73m^2^) (%)										
≥60	65.5(61.0-69.9)	40.3(36.0-44.5)	39.4(34.7-44.1)	35.5(31.0-40.0)	47.5(43.5-51.4)	41.1(37.3-44.9)	45.2(41.0-49.4)	44.4(40.4-48.3)	48.9(45.0-52.8)	48.6(44.8-52.5)
30-59	28.2(24.0-32.4)	52.7(48.3-57.0)	56.5(51.7-61.3)	58.1(53.5-62.7)	46.8(42.9-50.8)	51.1(47.2-54.9)	47.6(43.4-51.8)	49.4(45.4-53.3)	44.5(40.6-48.3)	44.5(40.7-48.3)
≤29	6.3(4.1-8.6)	7.1(4.8-9.3)	4.1(2.2-6.0)	6.4(4.1-8.7)	5.7(3.8-7.5)	7.8(5.7-9.8)	7.2(5.0-9.4)	6.3(4.4-8.2)	6.6(4.7-8.6)	6.9(4.9-8.8)
ACR (mg/g) (%)										
<30	12.6(9.5-15.8)	36.8 (32.6-41.1)	41.8 (37.0-46.6)	40.2 (35.5-44.8)	31.3 (27.6-35.0)	38.4 (34.7-42.2)	30.9 (27.0-34.9)	34.8 (31.0-38.6)	27.6 (24.1-31.2)	29.9 (26.3-33.5)
30-299	67.4(63.0-71.4)	50.6 (46.2-54.6)	48.8 (43.9-53.2)	49.1 (44.3-53.3)	55.0 (51.0-58.6)	50.0 (46.1-53.6)	55.2 (50.9-59.0)	52.2 (49.2-55.8)	55.4 (51.5-59.0)	55.5 (51.7-59.1)
≥300	19.9(16.1-23.7)	12.6 (9.6-15.5)	9.4 (6.6-12.3)	10.7 (7.8-13.7)	13.7 (11.0-16.5)	11.6 (9.1-14.0)	13.9 (1.0-16.9)	13.1 (10.4-15.8)	16.9 (13.9-19.9)	14.6 (11.8-17.3)

Numbers in table are expressed as column percent (95% confidence interval). Abbreviations: CKD, chronic kidney disease; CI: confidence interval; eGFR, estimated glomerular filtration rate; ACR, albumin-to-creatinine ratio; BMI, body mass index; BP, blood pressure; KDIGO, Kidney Disease Improving Global Outcomes. eGFR ≥60 mL/min/1.73m^2^ participants were defined as having CKD based on the presence of albuminuria; ACR <30 mg/g participants were defined as having CKD based on the decreased eGFR.

**Table 2:** The distribution of BP among adult CKD with hypertension from 1999-2000 to 2017-2018.

Characteristic	Calendar period									
	1999-2000 (n=443) (95% CI)	2001-2002 (n=509) (95% CI)	2003-2004 (n=414) (95% CI)	2005-2006 (n=437) (95% CI)	2007-2008 (n=617) (95% CI)	2009-2010 (n=644) (95% CI)	2011-2012 (n=540) (95% CI)	2013-2014 (n=620) (95% CI)	2015-2016 (n=632) (95% CI)	2017-2018 (n=654) (95% CI)
SBP/DBP category (%) ^#^										
<120/<80 (mmHg)	7.0(4.6-9.4)	9.8(7.2-12.4)	11.8(8.7-14.9)	14.0(10.7-17.2)	17.8(14.8-20.8)	17.4(14.5-20.3)	17.2(14.0-20.4)	19.8(16.7-23.0)	13.9(11.2-16.6)	14.7(12.0-17.4)
120-129/<80 (mmHg)	5.9(3.7-8.1)	8.4(6.0-10.9)	10.1(7.2-13.1)	15.3(12.0-18.7)	11.3(8.8-13.8)	14.8(12.0-17.5)	11.5(8.8-14.2)	12.9(10.3-15.5)	14.9(12.1-17.6)	11.8(9.3-14.2)
130-139/80-89 (mmHg)	14.2(11.0-17.5)	16.9(13.6-20.2)	18.1(14.4-21.8)	16.7(13.2-20.2)	17.5(14.5-20.5)	18.6(15.6-21.6)	21.1(17.7-24.6)	20.6(17.5-23.8)	17.1(14.2-20.0)	16.7(13.8-19.5)
140-159/90-99 (mmHg)	38.6(34.1-43.1)	36.5(32.4-40.7)	31.9(27.4-36.4)	33.9(29.4-38.3)	31.3(27.6-34.9)	32.6(29.0-36.2)	33.1(29.2-37.1)	27.3(23.8-30.8)	34.0(30.3-37.7)	34.4(30.8-38.0)
≥160/100 (mmHg)	34.3(29.9-38.7)	28.3(24.4-32.2)	28.0(23.7-32.3)	20.1(16.4-23.9)	22.0(18.8-25.3)	16.6(13.7-19.5)	17.0(13.9-20.2)	19.4(16.2-22.5)	20.1(17.0-23.2)	22.5(19.3-25.7)

^#^Adults were assigned to the higher SBP/DBP category when SBP and DBP levels that crossed over into another category. Numbers in table are expressed as column percent (95% confidence interval). Abbreviations: BP, blood pressure; CKD, chronic kidney disease; CI: confidence interval; SBP, systolic blood pressure; DBP, diastolic blood pressure.

**Table 3:** Percentage of uncontrolled BP among adult CKD with hypertension from 1999-2000 to 2017-2018.

Characteristic	Calendar									
	1999-2000(n=323) (95% CI)	2001-2002 (n=330) (95% CI)	2003-2004 (n=248) (95% CI)	2005-2006 (n=236) (95% CI)	2007-2008 (n=329) (95% CI)	2009-2010 (n=317) (95% CI)	2011-2012(n=271) (95% CI)	2013-2014 (n=289) (95% CI)	2015-2016 (n=342) (95% CI)	2017-2018 (n=372) (95% CI)
Overall	72.9(68.8-77.1)	64.8(60.7-69.0)	59.9(55.2-64.6)	54.0(49.3-58.7)	53.3(49.4-57.3)	49.2(45.4-53.1)	50.2(46.0-54.4)	46.6(42.7-50.5)	54.1(50.2-58.0)	56.9(53.1-60.7)
Age, y (%)										
20-44	7.1(4.3-9.9)	6.4(3.7-9.0)	4.8(2.2-7.5)	8.9(5.3-12.5)	4.9(2.5-7.2)	8.2(5.2-11.2)	6.6(3.7-9.6)	6.9(4.0-9.8)	9.4(6.3-12.4)	4.3(2.2-6.4)
45-64	21.1(16.6-25.5)	19.1(14.9-23.3)	23.0(17.7-28.2)	29.2(23.4-35.0)	34.3(29.2-39.5)	23.3(18.7-28.0)	29.2(23.7-34.6)	31.5(26.1-36.8)	29.2(24.4-34.1)	31.2(26.5-35.9)
65-74	28.2(23.3-33.1)	24.2(19.6-28.9)	28.6(23.0-34.3)	23.3(17.9-28.7)	24.6(20.0-29.3)	25.2(20.5-30.0)	23.2(18.2-28.3)	26.0(20.9-31.0)	21.9(17.5-26.3)	23.4(19.1-27.7)
≥75	43.7(38.2-49.1)	50.3(44.9-55.7)	43.5(37.4-49.7)	38.6(32.3-44.8)	36.2(31.0-41.4)	43.2(37.8-48.7)	41.0(35.1-46.8)	35.6(30.1-41.2)	39.5(34.3-44.7)	41.1(36.1-46.1)
Gender (%)										
Female	52.3(46.9-57.8)	52.1(46.7-57.5)	58.1(51.9-64.2)	49.6(43.2-56.0)	57.4(52.1-62.8)	48.9(43.4-54.4)	47.2(41.3-53.2)	50.2(44.4-55.9)	51.5(46.2-56.8)	52.4(47.3-57.5)
Race/ethnicity (%)										
Non-Hispanic White	41.8(36.4-47.2)	59.4(54.1-64.7)	54.0(47.8-60.2)	53.4(47.0-59.8)	47.4(42.0-52.8)	47.9(42.4-53.4)	39.5(33.7-45.3)	42.9(37.2-48.6)	35.7(30.6-40.7)	43.3(38.2-48.3)
Non-Hispanic Black	21.1(16.6-25.5)	20.9(16.5-25.3)	19.0(14.1-23.8)	28.8(23.0-34.6)	25.8(21.1-30.6)	21.1(16.6-25.6)	35.1(29.4-40.7)	29.1(23.8-34.3)	26.0(21.4-30.7)	25.3(20.9-29.7)
Mexican	30.0(25.0-35.0)	14.8(11.0-18.7)	19.0(14.1-23.8)	12.3(8.1-16.5)	13.7(10.0-17.4)	18.9(14.6-23.2)	5.5(2.8-8.3)	11.1(7.5-14.7)	17.5(13.5-21.6)	7.8(5.1-10.5)
Other	7.1(4.3-9.9)	4.8(2.5-7.2)	8.1(4.7-11.5)	5.5(2.6-8.4)	13.1(9.4-16.7)	12.0(8.4-15.6)	19.9(15.2-24.7)	17.0(12.6-21.3)	20.8(16.5-25.1)	23.7(19.3-28.0)
Education (%)										
<High school	54.2(48.7-59.6)	40.0(34.7-45.3)	39.1(33.0-45.2)	39.8(33.6-46.1)	42.9(37.5-48.2)	37.2(31.9-42.5)	41.3(35.5-47.2)	32.9(27.5-38.3)	33.3(28.3-38.3)	25.8(21.4-30.3)
High school graduate and some college	39.0(33.7-44.3)	47.3(41.9-52.7)	49.2(43.0-55.4)	49.2(42.8-55.5)	46.5(41.1-51.9)	48.6(43.1-54.1)	43.9(38.0-49.8)	50.2(44.4-55.9)	52.9(47.6-58.2)	57.3(52.2-62.3)
College graduate	6.8(4.1-9.6)	12.7(9.1-16.3)	11.7(7.7-15.7)	11.0(7.0-15.0)	10.6(7.3-14.0)	14.2(10.4-18.0)	14.8(10.5-19.0)	17.0(12.6-21.3)	13.7(10.1-17.4)	16.9(13.1-20.7)
Household income, $ (%)										
<44999	7.1(4.3-9.9)	6.4(3.7-9.0)	4.8(2.2-7.5)	8.9(5.3-12.5)	4.9(2.5-7.2)	8.2(5.2-11.2)	6.6(3.7-9.6)	6.9(4.0-9.8)	9.4(6.3-12.4)	4.3(2.2-6.4)
45000-74999	49.2(43.8-54.7)	43.3(38.0-48.7)	51.6(45.4-57.8)	52.5(46.2-58.9)	59.0(53.7-64.3)	48.6(43.1-54.1)	52.4(46.5-58.3)	57.4(51.7-63.1)	51.2(45.9-56.5)	54.6(49.5-59.6)
≥75000	43.7(38.2-49.1)	50.3(44.9-55.7)	43.5(37.4-49.7)	38.6(32.3-44.8)	36.2(31.0-41.4)	43.2(37.8-48.7)	41.0(35.1-46.8)	35.6(30.1-41.2)	39.5(34.3-44.7)	41.1(36.1-46.1)
Health insurance (%)										
None	11.8(8.3-15.3)	7.3(4.5-10.1)	7.3(4.0-10.5)	13.1(8.8-17.4)	12.2(8.6-15.7)	13.2(9.5-17.0)	14.0(9.9-18.2)	13.1(9.3-17.0)	13.2(9.6-16.7)	11.3(8.1-14.5)
Private	61.9(56.6-67.2)	52.7(47.3-58.1)	50.4(44.2-56.6)	90.3(86.5-94.0)	92.1(89.2-95.0)	92.7(89.9-95.6)	91.1(87.8-94.5)	90.7(87.3-94.0)	86.5(82.9-90.2)	92.7(90.1-95.4)
Medicare	71.8(66.9-76.7)	75.8(71.1-80.4)	78.2(73.1-83.4)	96.6(94.3-98.9)	95.4(93.2-97.7)	97.8(96.2-99.4)	95.9(93.6-98.3)	94.5(91.8-97.1)	95.6(93.4-97.8)	94.6(92.3-96.9)
government	5.9(3.3-8.4)	8.5(5.5-11.5)	10.5(6.7-14.3)	38.1(31.9-44.3)	38.0(32.7-43.2)	35.0(29.8-40.3)	37.6(31.9-43.4)	42.9(37.2-48.6)	43.9(38.6-49.1)	39.8(34.8-44.8)
Healthcare facility (%)	93.2(90.4-95.9)	96.1(94.0-98.2)	93.5(90.5-96.6)	92.4(89.0-95.8)	93.3(90.6-96.0)	92.4(89.5-95.3)	90.0(86.5-93.6)	92.0(88.9-95.2)	89.5(86.2-92.7)	90.9(87.9-93.8)
Had health care visit in past year (%)	93.2(90.4-95.9)	91.5(88.5-94.5)	92.7(89.5-96.0)	91.9(88.5-95.4)	90.9(87.8-94.0)	90.2(87.0-93.5)	87.5(83.5-91.4)	91.7(88.5-94.9)	86.8(83.3-90.4)	91.7(88.9-94.5)
Diabetes (%)	36.5(31.3-41.8)	34.2(29.1-39.4)	33.1(27.2-28.9)	36.4(30.3-42.6)	40.4(35.1-45.7)	40.1(34.7-45.5)	41.0(35.1-46.8)	40.8(35.2-46.5)	42.7(37.4-47.9)	43.8(38.8-48.9)
BMI (%)										
Normal	22.0(17.5-26.5)	26.4(21.6-31.1)	28.6(23.0-34.3)	21.6(16.4-26.9)	24.6(20.0-29.3)	23.0(18.4-27.7)	26.2(21.0-31.4)	22.8(18.0-27.7)	20.8(16.5-25.1)	19.4(15.3-23.4)
Overweight	34.7(29.5-39.9)	39.4(34.1-44.7)	37.9(31.9-43.9)	32.6(26.6-38.6)	34.7(29.5-39.8)	33.4(28.2-38.6)	28.8(23.4-34.2)	33.6(28.1-39.0)	36.5(31.4-41.7)	33.3(28.5-38.1)
Obese	43.3(37.9-48.7)	34.2(29.1-39.4)	33.5(27.6-39.3)	45.8(39.4-52.1)	40.7(35.4-46.0)	43.5(38.1-49.0)	45.0(39.1-50.9)	43.6(37.9-49.3)	42.7(37.4-47.9)	47.3(42.2-52.4)
Smoking status (%)										
Never smoked	46.4(41.0-51.9)	48.8(43.4-54.2)	51.6(45.4-57.8)	51.3(44.9-57.6)	50.8(45.4-56.2)	50.5(45.0-56.0)	54.2(48.3-60.2)	49.5(43.7-55.2)	49.1(43.8-54.4)	45.7(40.6-50.8)
Former smoker	35.0(29.8-40.2)	34.5(29.4-39.7)	37.9(31.9-43.9)	33.9(27.9-39.9)	33.4(28.3-38.5)	36.9(31.6-42.2)	36.5(30.8-42.3)	30.4(25.1-35.8)	32.7(27.8-37.7)	32.8(28.0-37.6)
Current smoker	11.5(8.0-14.9)	14.2(10.5-18.0)	13.7(9.4-18.0)	17.4(12.5-22.2)	17.3(13.2-21.4)	13.6(9.8-17.3)	17.7(13.2-22.3)	19.0(14.5-23.6)	16.4(12.5-20.3)	12.9(9.5-16.3)
eGFR (mL/min/1.73m^2^) (%)										
≥60	66.6(61.4-71.7)	44.8(39.5-50.2)	43.1(37.0-49.3)	39.4(33.2-45.6)	57.1(51.8-62.5)	47.6(42.1-53.1)	49.8(43.9-55.8)	48.8(43.0-54.6)	54.4(49.1-59.7)	55.9(50.9-61.0)
30-59	26.6(21.8-31.4)	49.7(44.3-55.1)	53.6(47.4-59.8)	51.7(45.3-58.1)	36.8(31.6-42.0)	46.1(40.6-51.5)	43.2(37.3-49.1)	43.9(38.2-49.7)	38.9(33.7-44.1)	35.8(30.9-40.6)
≤29	6.8(4.1-9.6)	5.5(3.0-7.9)	3.2(1.0-5.4)	8.9(5.3-12.5)	6.1(3.5-8.7)	6.3(3.6-9.0)	7.0(4.0-10.1)	7.3(4.3-10.3)	6.7(4.1-9.4)	8.3(5.5-11.1)
ACR (mg/g) (%)										
<30	9.6(6.3-12.9)	30.3 (25.3-35.4)	38.0 (31.9-44.1)	32.9 (26.8-39.0)	20.1 (15.7-24.5)	31.3 (26.2-36.4)	23.1 (18.0-28.2)	19.1 (14.9-23.3)	19.6 (15.4-23.7)	9.6 (6.3-12.9)
30-299	67.6(62.44-72.82)	54.2 (48.8-59.6)	50.4 (44.1-56.7)	56.3 (49.9-62.7)	61.4 (56.1-66.8)	52.2 (46.7-57.7)	58.3 (52.4-64.3)	59.2 (54.0-64.5)	64.3 (59.3-69.2)	67.6 (62.4-72.8)
≥300	22.8(18.1-27.4)	15.5 (11.5-19.4)	11.6 (7.5-15.6)	10.8 (6.8-14.8)	18.5 (14.2-22.8)	16.5 (12.4-20.5)	18.6 (13.9-23.3)	21.7 (17.3-26.1)	16.2 (12.4-20.0)	22.8 (18.1-27.4)

Numbers in table are expressed as column percent (95% confidence interval). Abbreviations: CKD, chronic kidney disease; CI: confidence interval; eGFR, estimated glomerular filtration rate; ACR, albumin-to-creatinine ratio; BMI, body mass index; BP, blood pressure; KDIGO, Kidney Disease Improving Global Outcomes. eGFR ≥60 mL/min/1.73m^2^ participants were defined as having CKD based on the presence of albuminuria; ACR <30 mg/g participants were defined as having CKD based on the decreased eGFR.

**Table 4:** Factors associated with uncontrolled BP among adult CKD with hypertension 2015-2018.

Characteristic	Adult CKD with hypertension (n=1506)			Adult CKD taking antihypertensive medication (n=1085)		
	Prevalence ratio %, (95% CI)			Prevalence ratio %, (95% CI)		
	Model 1	Model 2	Model 3	Model 1	Model 2	Model 3
Age, y						
20-44	1(ref)	1(ref)	1(ref)	1(ref)	1(ref)	1(ref)
45-64	0.58(0.29-1.07)	0.67(0.33-1.26)	0.80(0.38-1.54)	1.05(0.50-2.03)	1.19(0.60-2.53)	1.54(0.69-3.23)
65-74	0.66(0.32-1.24)	1.21(0.54-2.60)	1.81(0.77-4.08)	1.37(0.65-2.71)	1.59(0.79-3.48)	2.62(1.13-5.80)
≥75	0.79(0.39-1.48)	1.47(0.65-3.18)	**2.55(1.07-5.84)**	1.38(0.39-1.48)	1.80(0.88-3.85)	2.87(1.23-6.47)
Gender						
Female	1(ref)	1(ref)	1(ref)	1(ref)	1(ref)	1(ref)
Male	0.99(0.73-1.34)	1.01(0.74-1.39)	0.91(0.65-1.27)	0.95(0.69-1.30)	0.93(0.69-1.31)	0.96(0.66-1.38)
Race/ethnicity						
Non-Hispanic White	1(ref)	1(ref)	1(ref)	1(ref)	1(ref)	1(ref)
Non-Hispanic Black	**1.55(1.04-2.34)**	1.41(0.93-2.15)	1.30(0.85-2.02)	1.98(1.31-3.03)	**1.96(1.30-3.04)**	**1.93(1.23-3.09)**
Mexican	**2.02(1.31-3.20)**	**1.64(1.04-2.64)**	1.39(0.87-2.27)	2.14(1.37-3.43)	**1.87(1.20-3.07)**	**1.34(0.82-2.23)**
other	**1.88(1.14-3.23)**	1.31(0.76-2.35)	1.10(0.62-2.02)	1.77(1.05-3.09)	**1.30(0.76-2.41)**	0.96(0.53-1.81)
Education						
<High school	**1.71(1.12-2.68)**	**1.62(1.06-2.54)**	1.49(0.96-2.37)	1.66(1.08-2.62)	**1.58(1.03-2.51)**	**1.35(0.84-2.20)**
High school graduate and some college	1(ref)	1(ref)	1(ref)	1(ref)	1(ref)	1(ref)
College graduate	0.99(0.66-1.52)	1.19(0.78-1.86)	1.04(0.67-1.66)	1.01(0.67-1.56)	1.11(0.71-1.74)	0.84(0.53-1.36)
Household income, $						
≤44999	1(ref)	1(ref)	1(ref)	1(ref)	1(ref)	1(ref)
45000-74999	0.80(0.54-1.23)	0.86(0.56-1.33)	0.89(0.58-1.40)	0.83(0.54-1.29)	0.84(0.55-1.33)	0.83(0.52-1.35)
≥75000	**0.63(0.43-0.94)**	0.66(0.43-1.02)	0.72(0.46-1.14)	0.67(0.45-1.00)	0.72(0.47-1.11)	0.80(0.50-1.29)
Type of health insurance						
Private	0.71(0.50-1.00)	0.85(0.59-1.22)	0.90(0.62-1.31)	0.90(0.49-1.54)	0.96(0.52-1.66)	1.07(0.57-1.93)
Medicare	**0.52(0.32-0.84)**	**0.52(0.32-0.84)**	0.65(0.39-1.06)	1.01(0.45-2.04)	0.92(0.40-1.88)	1.19(0.51-2.53)
Government	0.88(0.57-1.41)	0.96(0.61-1.55)	1.01(0.63-1.64)	1.25(0.91-1.74)	1.29(0.94-1.81)	1(ref)
None	1(ref)	1(ref)	1(ref)	1(ref)	1(ref)	1.41(0.99-2.02)
Healthcare facility						
No	1(ref)	1(ref)	1(ref)	1(ref)	1(ref)	1(ref)
Yes	0.53(0.21-1.13)	0.54(0.22-1.16)	0.54(0.21-1.19)	0.64(0.25-1.39)	0.62(0.24-1.35)	0.66(0.25-1.49)
Healthcare visit in past year						
No	1(ref)	1(ref)	1(ref)	1(ref)	1(ref)	1(ref)
Yes	**0.28(0.08-0.72)**	**0.29(0.08-0.74)**	**0.33(0.10-0.87)**	**0.31(0.07-0.89)**	**0.31(0.07-0.92)**	**0.36(0.08-1.10)**
Diabetes						
No	1(ref)	1(ref)	1(ref)	1(ref)	1(ref)	1(ref)
Yes	1.11(0.81-1.53)	0.80(0.49-1.27)	0.98(0.70-1.39)	1.25(0.90-1.74)	0.74(0.45-1.20)	1.00(0.69-1.45)
BMI						
Normal	1(ref)	1(ref)	1(ref)	1(ref)	1(ref)	1(ref)
Overweight	0.94(0.57-1.51)	1.14(0.83-1.58)	1.02(0.61-1.68)	0.79(0.47-1.31)	1.24(0.90-1.73)	0.76(0.43-1.31)
Obese	0.74(0.46-1.17)	1.00(0.60-1.62)	0.88(0.53-1.42)	0.70(0.42-1.12)	0.80(0.47-1.33)	0.67(0.39-1.14)
Smoking status						
Never smoked	1(ref)	1(ref)	1(ref)	1(ref)	1(ref)	1(ref)
Former smoker	**0.21(0.03-0.71)**	**0.22(0.04-0.75)**	**0.25(0.04-0.88)**	0.75(0.52-1.08)	0.74(0.51-1.07)	0.78(0.52-1.17)
Current smoker	0.30(0.05-1.01)	0.31(0.05-1.06)	0.31(0.05-1.11)	0.63(0.40-1.01)	0.57(0.35-0.91)	**0.48(0.29-0.80)**
eGFR (mL/min/1.73m^2^)						
≥60	1(ref)	1(ref)	1(ref)	1(ref)	1(ref)	1(ref)
30-59	**0.28(0.19-0.40)**	**0.30(0.21-0.44)**	0.60(0.35-1.04)	**0.30(0.21-0.44)**	**0.31(0.21-0.46)**	**0.58(0.33-1.02)**
≤29	0.45(0.21-1.08)	0.52(0.24-1.24)	0.72(0.31-1.83)	0.66(0.31-1.58)	0.71(0.33-1.71)	1.19(0.46-3.56)
ACR (mg/g)						
<30	1(ref)	1(ref)	1(ref)	1(ref)	1(ref)	1(ref)
30-299	**3.95(2.77-5.67)**	**3.60(2.51-5.20)**	**2.25(1.39-3.75)**	**3.95(2.72-5.79)**	**3.87(2.65-5.69)**	**2.76(1.63-4.79)**
≥300	**4.39(2.53-8.06)**	**4.12(2.36-7.62)**	**3.14(1.71-6.07)**	**6.40(3.49-12.62)**	**6.07(3.29-12.02)**	**4.59(2.37-9.51)**

Numbers in table are expressed as prevalence ratio (95% confidence interval). Abbreviations: CKD, chronic kidney disease; CI: confidence interval; eGFR, estimated glomerular filtration rate; ACR, albumin-to-creatinine ratio; BMI, body mass index; BP, blood pressure; KDIGO, Kidney Disease Improving Global Outcomes. eGFR ≥60 mL/min/1.73m^2^ participants were defined as having CKD based on the presence of albuminuria; ACR <30 mg/g participants were defined as having CKD based on the decreased eGFR. Model 1: adjusted for age, sex, and race/ethnicity. Model 2: Model 1+ education, income, health insurance, healthcare facility and healthcare visit. Model 3: Adjusted for all characteristics listed.

## Abbreviations

ACC: American College of Cardiology
ACCORD: the Action to Control Cardiovascular Risk in Diabetes trial
ACR: albumin-to-creatinine ratio
AHA: American Heart Association
BMI: body mass index
BP: blood pressure
CI: confidence interval
CKD: chronic kidney disease
CVD: cardiovascular disease
DBP: diastolic blood pressure
eGFR: estimated glomerular filtration rate
ESRD: end stage of renal disease
JNC7: the Seventh Joint National Committee
JNC8: the Eighth Joint National Committee
KDIGO: Kidney Disease Improving Global Outcomes
NCHS: National Center for Health Statistics
NHANES: National Health and Nutrition Examination Survey
SBP: systolic blood pressure
SPRINT: the Systolic Blood Pressure Intervention Trial
