# Supplementary material for: Trends in Blood Pressure Control in US Adult CKD Patients from 1999 to 2018
Source: Int J Med Sci. 2025 Jan 13;22(3):696–707. doi: 10.7150/ijms.103107 (PMC11783084; doi:10.7150/ijms.103107)
Supplement: Supplementary file 1 — Supplementary figures and tables. [file ijmsv22p0696s1.pdf]

Supplementary Figure 1: Flowchart showing the selection of the study sample for current analysis, NHANES 1999-2018.

Calendar period

	Overall	1999-2000	2001-2002	2003-2004	2005-2006	2007-2008	2009-2010	2011-2012	2013-2014	2015-2016	2017-2018
Registered	101,317	9,965	11,039	10,123	10,348	10,149	10,537	9,756	10,175	9,971	9,254
SCr, ACR	63,970	6,230	6,891	6,492	6,434	6,376	6,860	5,976	6,553	6,255	5,903
CKD	10,684	879	1,129	1,066	1,144	1,161	1,099	1,025	1,109	1,017	1,055
≥20 years old	8,950	653	900	872	872	1,017	953	878	955	890	960
Not pregnant	8,876	643	882	867	849	1,017	950	871	950	888	959
3 times BP measurements	7,710	618	724	568	649	871	883	759	895	865	878
Had hypertension	5,510	443	509	414	437	617	644	540	620	632	654
Taking medication[#]	4,451	297	357	309	348	507	543	473	532	527	558

Abbreviations: SCr, serum creatinine; ACR, urinary albumin to creatinine ratio; BP, blood pressure; CKD, chronic kidney disease.

[#]Self-reported antihypertensive medication use.

Supplementary Figure 2. Proportion of CKD adult with hypertension awareness and antihypertensive medication use.

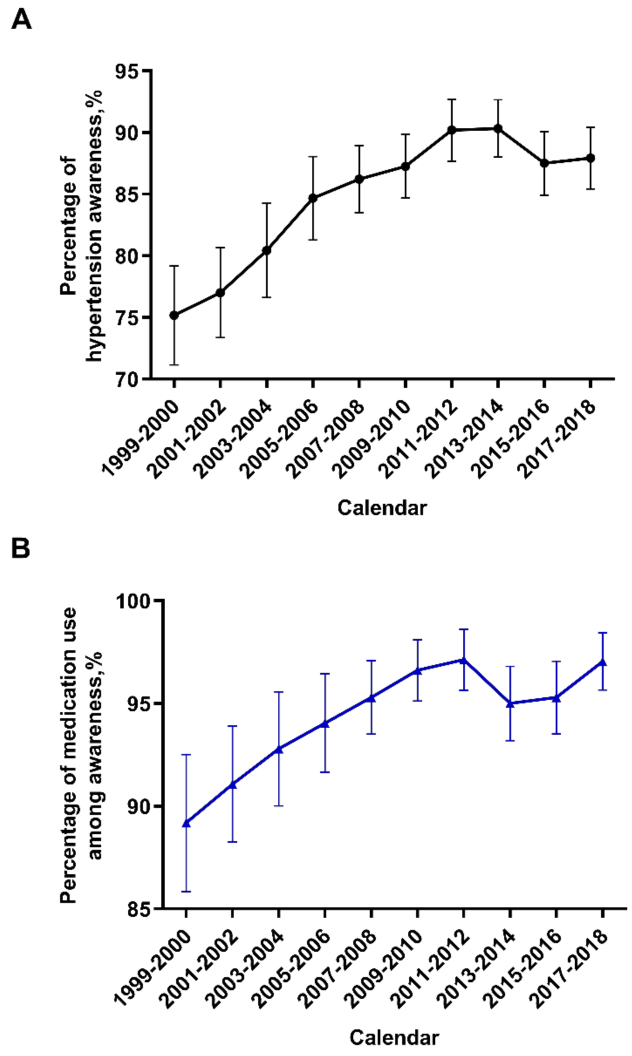

Error bars indicate 95% confidence intervals.

Supplementary Table 1. Variables in the current analysis and methods of ascertainment in the NHANES, 1999-2018.

Variable	Methods of ascertainment
Age	Self-report
Gender	Self-report
Race/ethnicity	Self-report
Education	Self-report
Type of health insurance	Self-report
Healthcare facility	Self-report
Healthcare visit in past year	Self-report
BMI	BMI examined at NHANES, BMI ≥30 is defined as obesity
Smoking status	Self-report
Diabetes	Fasting serum glucose ≥126 mg/dL, or non-fasting serum glucose ≥200 mg/dL, or glycosylated hemoglobin (HbA1c) ≥6.5% at NHANES examination, or self-reported use of glucose-lowing medication
Estimated glomerular filtration rate (eGFR)	Calculated by Chronic Kidney Disease Epidemiology Collaboration (CKD-EPI) equation based on serum creatinine measured at NHANES examination
Albumin-to-creatinine ratio (ACR)	Calculated by urinary albumin/urinary creatinine measured in spot urine sample measured at NHANES
Taking antihypertensive medication	self-reported use of antihypertensive medication
Hypertension awareness	self-reported that they have been told by a doctor or other health care professional that they had hypertension or high BP

Supplementary Table 2. Factors associated with hypertension awareness and taking antihypertensive medication among US adults with hypertension in 2015-2018.

Characteristic	Adult CKD with hypertension awareness (n=1,286)<br>Prevalence ratio %, (95% CI)			Adult CKD taking antihypertensive medication among those who were aware of hypertension (n=1,128)<br>Prevalence ratio %, (95% CI)		
	Model 1	Model 2	Model 3	Model 1	Model 2	Model 3
**Age, y**						
20-44	1(ref)	1(ref)	1(ref)	1(ref)	1(ref)	1(ref)
45-64	1.62(0.85-2.97)	1.18(0.59-2.28)	1.05(0.51-2.08)	**3.41(1.51-7.46)**	**2.47(1.01-5.85)**	2.14(0.82-5.42)
65-74	**2.39(1.20-4.61)**	1.52(0.72-3.09)	1.29(0.59-2.77)	**6.31(2.52-16.31)**	**4.26(1.54-12.01)**	2.68(0.90-8.10)
≥75	1.47(0.77-2.69)	0.92(0.45-1.81)	0.90(0.41-1.92)	**18.04(5.85-68.08)**	**11.50(3.38-46.33)**	**4.59(1.21-20.19)**
**Gender**						
Female	1(ref)	1(ref)	1(ref)	1(ref)	1(ref)	1(ref)
Male	0.93(0.66-1.31)	0.89(0.62-1.26)	0.80(0.55-1.17)	0.67(0.34-1.27)	0.69(0.35-1.35)	0.73(0.36-1.49)
**Race/ethnicity**						
Non-Hispanic White	1(ref)	1(ref)	1(ref)	1(ref)	1(ref)	1(ref)
Non-Hispanic Black	1.58(0.99-2.59)	1.55(0.96-2.57)	1.49(0.91-2.48)	1.66(0.67-4.38)	1.42(0.55-3.85)	1.50(0.57-4.21)
Mexican	1.08(0.68-1.72)	1.17(0.67-2.12)	1.35(0.75-2.50)	0.78(0.34-1.79)	0.64(0.23-1.84)	0.54(0.19-1.61)
other	0.82(0.49-1.38)	1.36(0.84-2.26)	1.58(0.95-2.69)	0.67(0.27-1.71)	0.75(0.31-1.83)	0.73(0.29-1.86)
**Education**						
<High school	**0.62(0.42-0.94)**	**0.64(0.42-0.99)**	**0.64(0.41-0.98)**	1.26(0.60-2.81)	1.48(0.67-3.48)	1.49(0.66-3.56)
High school graduate and some college	1(ref)	1(ref)	1(ref)	1(ref)	1(ref)	1(ref)
College graduate	1.07(0.65-1.83)	0.96(0.57-1.69)	1.11(0.64-1.97)	2.18(0.81-7.63)	1.68(0.58-6.16)	1.68(0.57-6.25)
**Household income, $**						
≤44999	1(ref)	1(ref)	1(ref)	1(ref)	1(ref)	1(ref)
45000-74999	1.03(0.66-1.68)	1.02(0.64-1.69)	1.02(0.63-1.69)	**3.32(1.15-14.09)**	2.91(1.00-12.39)	2.42(0.80-10.58)

≥75000	1.24(0.77-2.06)	1.06(0.64-1.83)	1.04(0.61-1.83)	2.56(0.99-8.76)	2.30(0.83-8.22)	1.89(0.65-7.01)
**Type of health insurance**						
Private	0.71(0.34-1.34)	0.70(0.33-1.34)	0.67(0.31-1.29)	0.37(0.06-1.28)	0.37(0.06-1.28)	0.31(0.05-1.13)
Medicare	0.55(0.16-1.39)	0.59(0.17-1.52)	0.62(0.18-1.63)	0.32(0.02-1.58)	0.39(0.02-1.99)	0.47(0.03-2.49)
Government	1.19(0.84-1.69)	1.08(0.76-1.55)	1.07(0.74-1.54)	**0.53(0.27-0.99)**	**0.47(0.24-0.93)**	**0.43(0.21-0.86)**
None	1(ref)	1(ref)	1(ref)	1(ref)	1(ref)	1(ref)
**Healthcare facility**						
No	1(ref)	1(ref)	1(ref)	1(ref)	1(ref)	1(ref)
Yes	**2.64(1.57-4.34)**	1.47(0.79-2.62)	1.41(0.75-2.56)	**3.29(1.47-6.98)**	**2.62(1.04-6.26)**	2.28(0.86-5.79)
**Healthcare visit in past year**						
No	1(ref)	1(ref)	1(ref)	1(ref)	1(ref)	1(ref)
Yes	**4.87(2.94-8.01)**	**4.03(2.28-7.08)**	**3.77(2.09-6.77)**	**3.03(1.24-6.85)**	2.27(0.82-5.88)	2.32(0.80-6.35)
**Diabetes**						
No	1(ref)	1(ref)	1(ref)	1(ref)	1(ref)	1(ref)
Yes	**1.57(1.10-2.27)**	**1.52(1.05-2.21)**	**1.34(0.82-2.17)**	1.50(0.77-3.01)	1.56(0.77-3.24)	0.69(0.22-1.94)
**BMI**						
Normal	1(ref)	1(ref)	1(ref)	1(ref)	1(ref)	1(ref)
Overweight	1.37(0.86-2.16)	1.32(0.82-2.11)	**1.69(1.03-2.77)**	0.94(0.32-2.46)	0.88(0.28-2.40)	0.73(0.24-1.99)
Obese	**1.84(1.16-2.88)**	**1.71(1.06-2.73)**	1.46(0.98-2.17)	1.22(0.43-2.99)	1.07(0.36-2.76)	1.38(0.65-3.02)
**Smoking status**						
Never smoked	1(ref)	1(ref)	1(ref)	1(ref)	1(ref)	1(ref)
Former smoked	1.37(0.93-2.04)	1.33(0.89-2.00)	1.30(0.87-1.97)	0.92(0.39-2.24)	0.93(0.38-2.33)	0.86(0.34-2.20)
Current smoked	**2.05(1.17-3.83)**	**2.31(1.28-4.42)**	**2.68(1.47-5.17)**	**0.28(0.13-0.59)**	**0.31(0.13-0.68)**	**0.33(0.14-0.74)**
**eGFR (mL/min/1.73m^2)**						

≥60	1(ref)	1(ref)	1(ref)	1(ref)	1(ref)	1(ref)
30-59	**1.61(1.10-2.39)**	**1.51(1.01-2.26)**	1.32(0.78-2.32)	**3.58(1.52-9.94)**	**3.63(1.49-10.25)**	**7.43(1.42-13.7)**
≤29	**11.05(2.37-19.7)**	**9.65(2.05-17.2)**	**8.53(1.75-15.4)**	1.70(0.24-4.20)	2.10(0.31-4.30)	1.20(0.15-3.46)
**ACR (mg/g)**						
<30	1(ref)	1(ref)	1(ref)	1(ref)	1(ref)	1(ref)
30-299	0.66(0.43-1.01)	0.71(0.46-1.10)	0.82(0.45-1.52)	0.44(0.14-1.07)	0.47(0.15-1.20)	2.80(0.41-55.84)
≥300	0.84(0.47-1.52)	0.89(0.49-1.65)	0.85(0.43-1.75)	1.21(0.30-5.17)	1.44(0.35-6.36)	5.37(0.68-118.88)

Numbers in table are expressed as prevalence ratio (95% confidence interval).

Abbreviations: CKD, chronic kidney disease; CI: confidence interval; eGFR, estimated glomerular filtration rate; ACR, albumin-to-creatinine ratio; BMI, body mass index; BP, blood pressure.

eGFR ≥60 mL/min/1.73m^2 participants were defined as having CKD based on the presence of albuminuria; ACR <30 mg/g participants were defined as having CKD based on the decreased.

Model 1: adjusted for age, sex, and race/ethnicity.

Model 2: Model 1+ education, income, health insurance, healthcare facility and healthcare visit.

Model 3: Adjusted for all characteristics listed.
